# Understanding hip pathology in ballet dancers

**DOI:** 10.1007/s00167-022-06928-1

**Published:** 2022-03-19

**Authors:** Yash Singh, Matthew Pettit, Osama El-Hakeem, Rachel Elwood, Alan Norrish, Emmanuel Audenaert, Vikas Khanduja

**Affiliations:** 1 Addenbrooke’s Hospital, University of Cambridge School of Clinical Medicine, Hills Road, Cambridge, CB2 0SP UK; 2 Academic Orthopaedics, Trauma and Sports Medicine, University of Nottingham, Queens Medical Centre, Nottingham, NG7 2UH UK; 3 Ghent University Hospital, C. Heymanslaan 10, Ingang 46-Verdieping 4, 9000 Ghent, Belgium; 4 Department of Trauma and Orthopaedics, Young Adult Hip Service, Addenbrooke’s-Cambridge University Hospital, Box 37, Hills Road, Cambridge, CB2 0QQ UK

**Keywords:** Ballet, Dancer, Hip, Injury, Pathology, Prevalence, FAI, Dysplasia, Outcomes.

## Abstract

**Purpose:**

The literature on hip injuries in ballet dancers was systematically evaluated to answer (1) whether the prevalence of morphological abnormalities and pathology of hip injuries in dancers differs from the general population (2) if there are any specific risk factors which contribute to a higher rate of hip injury and (3) what are the outcomes of primary and secondary intervention strategies.

**Methods:**

A systematic literature search of Medline, EMBASE and the Cochrane Library was undertaken for all literature relating to hip injuries in ballet dancers using the PRISMA guidelines. Reference lists were also searched for relevant literature. Clinical outcome studies, prospective/retrospective case series published between 1989 and October 2021 were included. Review articles (non-original data), case reports, studies on animals as well as book chapters were excluded.

**Results:**

The search yielded 445 studies, of which 35 were included for final analyses after screening. This included 1655 participants, of which 1131 were females. The analyses revealed that damage at the chondrolabral junction and degenerative disease of the hip may develop at a higher rate in ballet dancers than in the general population (odds ratio > 1 in 15/18 cohorts). The intra-articular lesions were more frequently found in postero-superior region of the hip suggesting an alternative impingement mechanism. Furthermore, numerous risk factors specific for hip injury in ballet were highlighted amidst a wide body of literature which consistently reports risk factors for a more generic ‘dancer vulnerability’.

**Conclusion:**

Ballet dancers may suffer from both higher rates of chondrolabral damage and degenerative disease in their hips. In contrast to other sports, the intra-articular lesions are more frequently found in postero-superior region of the hip. Future research clarifying the prevalence of osseous abnormalities and prevention strategies in dancers may be pivotal in delaying the development of hip disease in this cohort.

**Level of evidence:**

Level IV.

## Introduction

Ballet is a traditional and highly technical form of dance which began in Italy before its export to France and Russia where it prospered during the Renaissance period [38]. The discipline combines athletic expertise with art to incite emotion in its audience. Dancers usually begin training at a very young age with males and females tending to take on more athletic and technical components of dance pieces, respectively [72].

Professional dance companies report that as many as 67–95% of their dancers are injured on annual basis [24]. Similarly, an injury incidence of 1.1 injuries per dancer per annum has been described in a 10-year retrospective study [63]. Ballet dancers take on a high athletic load. Dancers typically perform over 200 jumps during a class, the majority of which are landed unilaterally, exposing their lower limbs to ground reaction forces as high as nine times their body weight [20, 46]. A systematic review found dancers to suffer from a high rate of hip injury at 17.7%, of which 9 of 13 cohorts were ballet dancers alone [76]. In the selected cohorts, the incidence of hip injury was 0.09 per 1000 h. Another retrospective study found that 21.6% of injuries in elite adolescent ballet dancers occurred at the hip [24]. Seventy-five percent of injuries were overuse or non-traumatic in nature [69] with many aetiological factors proposed, including supra-physiological demands, extreme ranges of motion, improper technique, dance-specific biomechanics [34], morphological abnormalities and poor strength and conditioning. In addition to the short term consequences, repetitive injury predisposes dancers to long-term pain [67, 70], disability [67], a decreased quality of life [28] and increased rates of hip osteoarthritis (OA) [3, 16].

The combination of risk factors is unique to ballet. As such, the underlying pathology and consequent management of the ballet dancer’s hip requires a personalised approach. Primary prevention strategies recognise and alleviate risk factors for hip injury. Secondary and tertiary prevention strategies aim to delay disease onset and severity. They must include a comprehensive approach to the dancer’s injury, appreciating the unique demands of dance and consequent hip pathology. It has been assumed that correcting range of motion (ROM) limiting morphological abnormalities (impingement, dysplasia, version, ligament, and muscular tightness) would allow resumption of athletic activity, however, impingement characteristics and the specific requirements of joint motion vary greatly across different sports. In ballet dancers, it has been reported that impingement and degenerative change is likely to occur through supra-physiological range ROM, rather than aberrant bony morphology, although ROM limiting factors have been suggested to further vary between dancers [32].

For these reasons, the literature regarding hip injuries in ballet dancers was systematically evaluated to answer (1) whether the prevalence of morphological abnormalities and pathology of hip injuries in dancers differs from the general population (2) if there are any specific risk factors which contribute to a higher rate of hip injury and (3) what are the outcomes of primary and secondary intervention strategies. This may aid in the development of intervention strategies targeted towards the unique risk factors and pathology seen in the hips of ballet dancers.

## Methods

### Study design

A scoping review was designed based on the methodological frameworks outlined by Arksey and O’Malley [4] and advanced by others [13, 45]. PRISMA [78] and the Joanna Briggs Institute [61] guidelines were similarly followed.

### Eligibility criteria

Clinical outcome studies, prospective/retrospective case series published between 1989 and October 2021 were included. Review articles (non-original data), case reports, studies on animals as well as book chapters were excluded. During the screening process, articles not specific to hip injury or ballet dance were excluded. Similarly, studies describing biomechanics with no reference to pathology were excluded.

### Search strategy

**Fig. 1:**
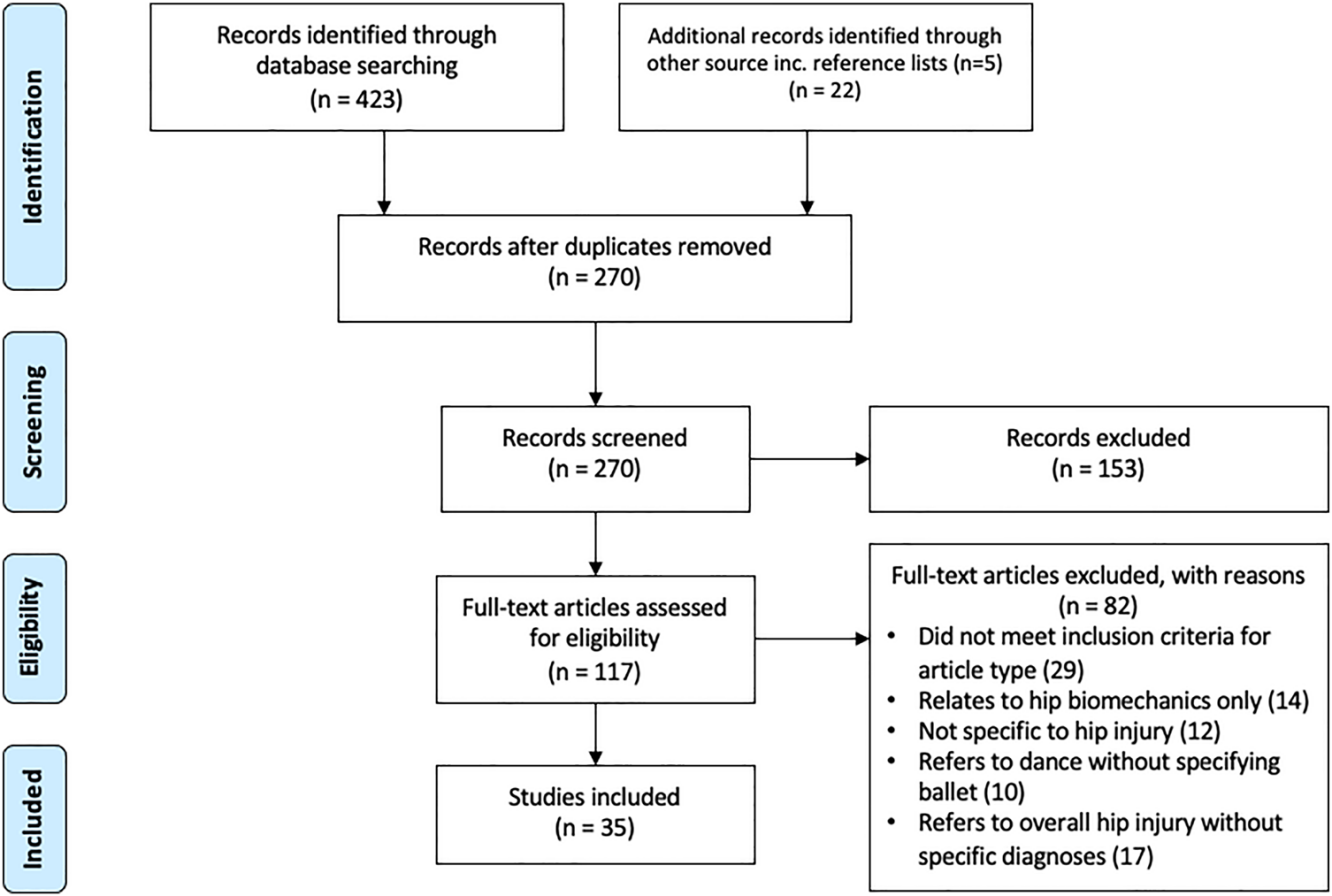
The search processes

A computer-assisted search of Embase, MEDLINE and the Cochrane Library for articles related to hip injuries in ballet dancers was completed on the 11th of October 2021 using the search terms “hip” and “ballet or ballerina”. The process for screening is detailed in Fig. 1 and the search strategy breakdown in Table 1. Two independent reviewers (YS and MP) completed the screening process, individually and blinded from one another, with any disagreements resolved by a third reviewer (VK).

### Data extraction

All included studies were charted by two independent reviewers (YS and MP) and then discussed for synthesis. Data were extracted and summarised on Microsoft Excel using a template which reflected the study objectives. The extracted data included the key characteristics of the studies including the study authors, year of publication, population, design, age, sample size, hip-specific aetiological factors, prevalence of pathology and clinical outcomes.

### Comparison of prevalence

Where possible, the prevalence of hip pathology in ballet dancers was compared to non-athletic controls to appreciate the hip pathology that the ballet dancers are pre-disposed to. This was possible where the study itself included a non-athletic control or where the prevalence was reported for similar populations in the literature. The control and population prevalence values were compared to values in ballet populations in order to determine an odds ratio for the development of a given pathology and given ballet participation [14, 15, 17, 23, 26, 27, 32, 36, 44, 62, 65, 77, 81]. This was not possible for the reported values of certain hip injury diagnoses due to the lack of comparative controls in the literature.

## Results

The search yielded 445 studies, of which 35 were included for final analyses after screening. This included 1655 participants, of which 1131 were females (Fig. 1). Thirty-four of the included studies were observational, whilst one was of an in silico design.

### Prevalence

**Fig. 2:**
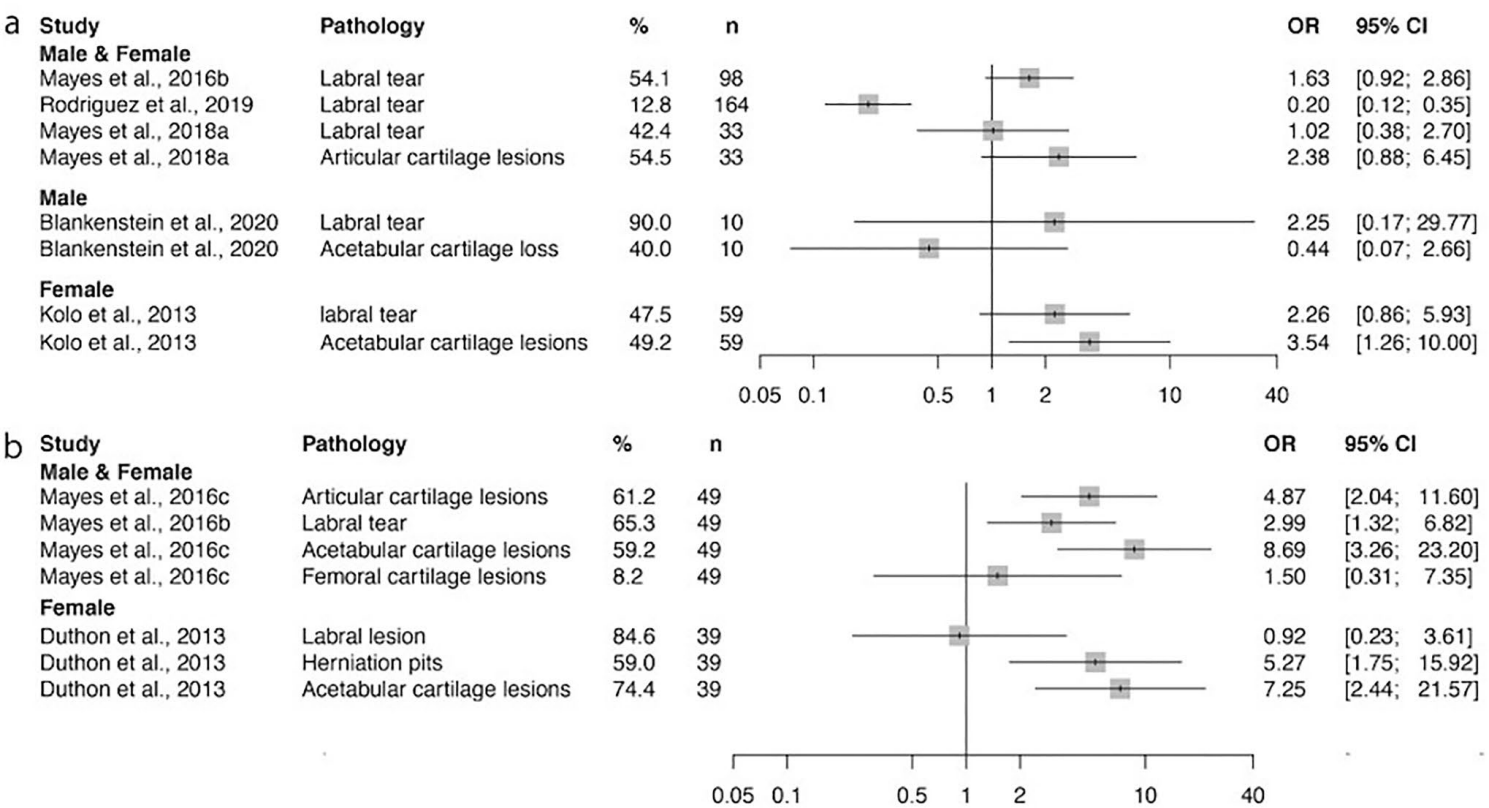
The prevalence of hips with damage at the chondrolabral junction (including articular lesions and labral tears). Odds ratio and confidence interval values for individual studies given by comparing these values with those in the general population. Prevalence measured **a** per hip and **b** per person. Chondrolabral damage at the hip joint seems to occur at a higher rate in ballet dancers than in the general population

**Fig. 3:**
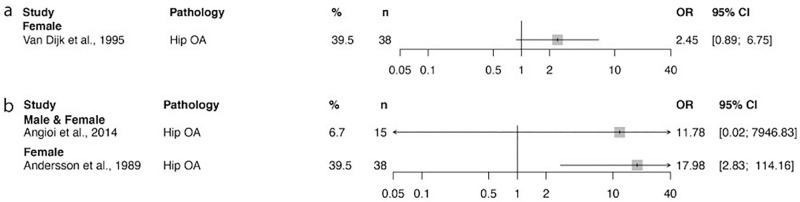
The prevalence of degenerative disease of the hip in ballet. Odds ratio and confidence interval values for individual studies given by comparing these values with those in the general population. Prevalence measured **a** per hip and **b** per person. Degenerative disease at the hip joint seems to occur at a higher rate in ballet dancers than in the general population

**Fig. 4:**
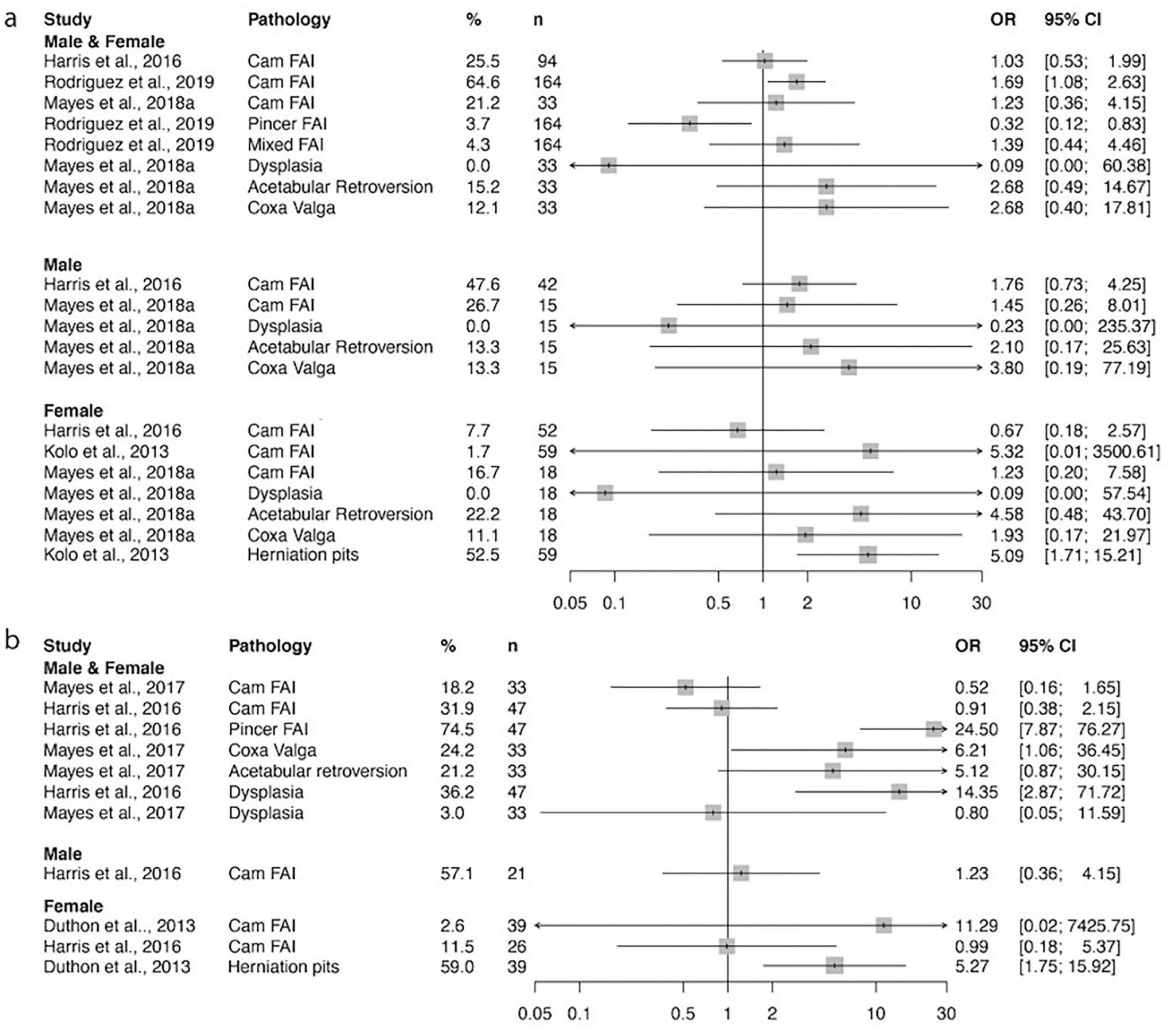
**a** The prevalence of osseous abnormalities in ballet dancers’ hips. Odds ratio and confidence interval values of individual studies given by comparing these values with those of the general population. Prevalence measured **a** per hip and **b** per person. Osseous abnormalities at the hip joint occurs at a similar rate in ballet dancers than in the general population

The prevalence of degenerative hip pathology, osseous abnormalities and of specific hip injuries were recorded. This was compared to the prevalence of hip disease within the general population for the study populations displayed in Figs. 2, 3 and 4. Damage at the chondrolabral junction as well as degenerative disease appears to have a higher prevalence in ballet dancers than in the general population (Figs. 2, 3).

The prevalence of osseous abnormalities which may act to predispose to degenerative disease is reported in Fig. 4. Additionally, borderline dysplasia (LCEA 20°–25°) was reported at a high prevalence of 15–53% [33, 36, 44, 49, 54, 55]. Femoral version was also investigated in three studies. One study measured version using MRI which did not differ to femoral version in the general population [6], whilst the other studies assessed version using ultrasound or an inclinometer and did not include controls [29, 30].

The incidence of injuries sustained in ballet was reported both as point prevalence and as incidence per 1000 dance hours. Point prevalence is presented in Table 2. The prevalence of ligamentum teres injuries (55%) was higher than what tends to be reported for the general population [49], and higher than athletic controls who participate in tennis, netball or basketball(*p* = 0.001) [54]. The prevalence of hip joint effusion-synovitis was higher than in controls who participate in tennis, netball or basketball [50]. The prevalence of iliopsoas snapping was also higher than estimated within the general population [82]. The lack of wider population studies made it difficult to compare the incidence of injury per 1000 dance hours, which is presented in Table 3 [2, 43, 72].

### Risk factors for hip injury in ballet

Risk factors specific to hip injury in ballet dancers are displayed in Table 4 [9]. Important factors which may have no effect on injury included generalised joint hypermobility [52–54, 57], BMI and the strength of the external rotators [21] and both obturator internus and externus [51]. Factors which have been reported to have an effect on hip injury include extreme ranges of motion and subluxation episodes leading to impingement and degenerative disease [5, 11, 19, 39]. The presence of impingement-type osseous morphology including cam and/or pincer morphology, low neck shaft angle (NSA < 125°) and acetabular version < 10° or > 20° also contributed to degenerative disease [56, 58, 59]. Hip pain was associated with reduced iliopsoas strength [22], low alpha angles [7], and female sex [72]. Increasing age was associated with ligamentum teres tears and degenerative hip disease, but also a lower rate of snapping hip. Finally, ballet as a discipline in itself influenced the frequency and location of soft tissue hip injury [73].

### Outcomes for treatment of hip pathology in ballet dancers

The outcomes for specific interventions are displayed in Table 5. Additionally, two studies reported on the effect of previous self-reported hip injury on ballet dancers’ current quality of life. Gross et al. [28] reported a decreased HOOS QoL score (*p* = 0.0001), whilst Biernacki et al. [8] reported a significant negative correlation between iHOT-12 scores and the total number of past hip injuries.

## Discussion

The most important finding of the present study was that that damage at the chondrolabral junction and degenerative disease of the hip may develop at a higher rate in ballet dancers than in the general population. Second, in contrast to other sports, the intra-articular lesions are more frequently found in postero-superior region of the hip. Snapping syndromes of the hip, effusion-synovitis and ligamentum teres injuries are also highly prevalent in ballet dancers. The data regarding FAI and dysplasia is more heterogenous and less consistent, requiring further evaluation. The concept of micro-instability and hip impingement-subluxation has been widely proposed and may be considered as an antecedent and consequence of other hip pathologies.

Numerous risk factors specific for hip injury in ballet were identified, amidst a wide body of literature which consistently reports risk factors for a more generic ‘dancer vulnerability’. This is an important step towards introducing preventative strategies for hip disease in dancers. With regards to outcomes, a 100% return to dance was described in conservative management of snapping hip [42], and a high rate was also described after peri-acetabular osteotomy [60] (PAO: 63%) and arthroscopy [80] (97%).

### Degenerative disease

The consequences of hip OA are devastating, both functionally and economically. Studies reported both increased rates of chondrolabral junction damage (including ‘labral tears’, ‘cartilage lesions’, ‘articular cartilage lesions’) and of end-stage degenerative disease (Figs. 2, 3). The odds ratio was greater than one for 12/15 and 3/3 cohorts, respectively. As labral tears and articular cartilage lesions form a single layer which is likely to be damaged concurrently, the data were combined to form the chondrolabral junction. Damage to this layer may represent an early stage in the subsequent development of degenerative hip disease.

The only longitudinal study available, however, concluded that in the majority of dancers, cartilage defects do not progress over 5 years [58]. Despite this, those with cartilage lesions do become symptomatic albeit with participation being affected minimally. A further study with larger population sizes and longer follow-up would help clarify how the degenerative process develops and how it is exacerbated by ballet.

### Osseous abnormalities

The incidence of osseous abnormalities such as FAI or dysplasia, however, is more variable and further investigation is required for definitive conclusions (Fig. 4). Despite this, dancers with FAI seem to suffer from greater rates of subluxation, instability, and pain. Where studies did not report a matched control population, the ballet population prevalence was compared to the prevalence reported in non-sporting populations within the literature. Future studies will benefit from matching ballet dancers with non-athletic controls for accurate comparison and determination of aetiology.

Bony abnormalities such as dysplasia may enhance the dancer’s ROM despite simultaneously decreasing hip-joint stability and predisposing the dancer to hip injury and early onset OA. Conversely, abnormalities which limit hip ROM such as FAI may exacerbate abutment between the femoral head–neck junction and the acetabular rim, thereby decreasing joint mobility. FAI [25, 35] and dysplasia [1, 75] have both been shown to increase the risk of osteoarthritis in athletic and general populations [83]. In ballet dancers, impingement-type morphology was related to cartilage defects [56] in one study and related to both labral tears and instability in another [66].

**Fig. 5:**
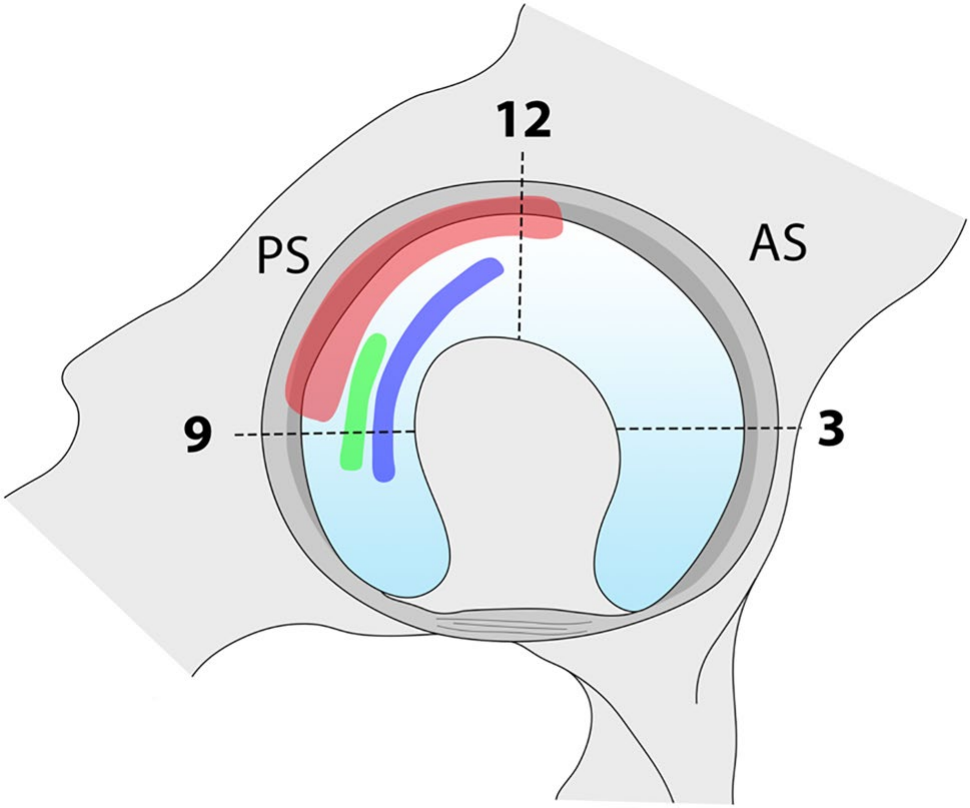
Schematic diagram indicating the postero-superior impingement identified across four studies. Red: this area represents the position of cartilage damage reported by Duthon et al., Kolo et al., and Charbonnier et al., Green: this area represents peak compression forces identified by Assassi and Thalman using in silico modelling of ballet hip movements. Blue: this area represents the location of impingements modelled to occur in extremes of motion achieved in ballet by Charbonnier et al. and Assassi and Thalman

Whilst it is mechanistically attractive to attribute functional impairment and degenerative disease to these bony abnormalities, hip instability can be both an antecedent and consequence of other hip pathologies in the ballet population. In a professional ballet company, 89% of dancers had hip subluxation, 36% of which broke the suction seal of the hip joint [59]. In all movements, subluxation accompanied impingement highlighting the contribution of bony morphology in exacerbating instability related pathology. An association between impingement and micro-instability has been shown using ultrasound scans [66] and MRI [12]. Interestingly, impingement zones were located at the superior and postero-superior areas of the acetabulum which corresponds to the diagnosed damaged areas in the labrum. Furthermore, all of these hips were morphologically normal. Kolo et al. [39] and Duthon et al. [19] both illustrated similar findings with MRI reporting subluxation and a high prevalence of supero-posterior chondrolabral injury, without evidence of cam or pincer morphologies. It has therefore been theorised that intermittent subluxation induced incongruency may instigate an early degenerative process in the dancers’ hip. As such, the pathogenesis of FAI in ballet dancers seems to differ from that in other sporting populations, with a subluxation-impingement-type injury occurring which may be exacerbated by abnormal bony morphology. The chondral and labral pathology occurs in the postero-superior position of the hip, in comparison to the antero-superior position commonly observed in non-dancing athletes. The finite element modelling of Assassi et al. [5] provides further weight to this theory, evidencing cartilage hyper-compression in the postero-superior positions of the hip during extreme ROMs in ballet. These forces reflect the impinging hip identified in earlier studies and act as a mechanism for recurrent microtrauma during dance, ultimately leading to degenerative hip disease (Fig. 5).

Other causes of hip instability are also likely to play a role in the development of OA. In ballet dancers, a higher frequency of ligamentum teres tears are found in comparison to non-dance athletes (55%, *p* = 0.001) [54] and isolated LT tears have been associated with premature OA [64] and hip pain [10]. It is, however, unknown whether there is a role of other osseous factors, such as version, in contributing to hip instability and long-term degeneration in ballet dancers, and current suggestions are speculative.

### Risk factors for hip injury

One of our studies’ main objectives was to investigate the ballet dancers’ vulnerability to hip injury. Many specific risk factors are presented in Table 4 although no clear patterns emerge, except for the subluxation-impingement mechanism described above. There is, however, a lack of clarity between specific risk factors for hip injury and risk factors for overall injury, or dancer vulnerability, throughout the literature. This distinction is important as the studies which focused on risk factors for overall injury yielded no quantitative data for our analysis. Some important risk factors identified for overall injury are discussed.

The majority of dance injuries are overuse, highlighting a lack of recovery in the training regimes of ballet dancers. Liederbach et al. [47] found that for dancers reporting injuries, 90% were “feeling tired at time of injury,” and roughly 80% were during high intensity work or when they had been dancing for more than five hours. Matters relating to fatigue such as training duration, hours, intensity, seasonal/transition times have all been associated with injury and so Lin et al. [48] propose that fatigue impairs muscle output and postural control, both of which increase the risk of injury. This risk may be exacerbated by factors such as inadequate strength and conditioning. Indeed, a study by Koutedakis et al. [41] noted that muscle flexibility, anaerobic power, and leg strength actually increased during a period of rest. In addition, Twitchett et al. found [79] that dancers with a lower level of fitness suffered from more injuries. Dancers may benefit from a more functional approach to strength and conditioning as dance training may not build a strong aerobic foundation in comparison to other sports [40, 68]. For example, a core strengthening program was shown to improve several fitness parameters such as jumping, proprioception, co-ordination and dynamic balance [37]. Similarly, a wider approach to the health of ballet dancers may help prevent injury as dysfunctional eating behaviour and/or menstrual abnormalities may contribute to injury and poor recovery. Dancers, especially ballerinas, have a higher prevalence of RED-S (formerly female athlete triad) than many other sports [18, 71].

In addition to the subluxation-impingement-type injury, extreme ranges of motion may push dancers to employ compensatory mechanisms along their kinetic chain. For example, the lack of a perfect turnout can result in overpronation (“rolling”), increased lumbar lordosis and torsion (“screwing”) at the knees. Extreme ranges of motion can also result in soft tissue adaptations and laxity which whilst perhaps initially being protective may eventually allow greater stress to be placed on the hip joint such as during subluxation episodes [19, 31, 39, 74].

### Outcome of preventative strategies

Very few studies have investigated the efficacy of preventative strategies and return to dance in ballet populations. Sixty-three percent of young female dancers with dysplasia returned to dance after PAO. There was an overall improvement in their pain, sports-related and daily activities, and hip function assessed by the HOOS and the mHHS [60]. A high return to dance (97%) was also evident after hip arthroscopy with 63% returning to a better level of participation. Statistically significant increases were observed for HOOS and mHHS [80]. It is important to note that the cohort was predominantly female with, at most, borderline dysplasia, and no radiographic evidence of hip OA. The careful selection of patients with a treatable cam lesion and without significant joint laxity or dysplasia may be critical to ensuring good patient outcomes [12]. Similarly, in a mixed cohort of dancers, all returned to dance after conservative management for the treatment of iliopsoas syndrome [42]. Future study investigating the efficacy of preventative strategies on hip injury specifically are required to best guide future practise. Similarly, further work identifying and alleviating specific risk factors such as strength or core training for muscular imbalances may enable healthcare professionals to prevent hip injury in ballet dancers.

In addition to limitations already discussed, our scoping review included a wide variety of study designs and thus, the level of evidence was not constant. Additionally, a significant proportion of the literature is based on a small number of subjects who are reported on across numerous studies. Due to the heterogeneity of current studies, we were unable to perform a systematic review and meta-analysis of the prevalence of degenerative disease, bony abnormalities, or other hip pathology in ballet dancers. Similarly, the number of subjects with certain pathologies, such as hip OA, were low. Studies prior to 1989 were excluded due to the paucity of literature prior to this year.

## Conclusion

Ballet dancers are a unique sporting population who combine artistry with athleticism. This study shows that ballet dancers may suffer from both higher rates of chondrolabral damage and degenerative disease in their hips. The intra-articular lesions are more frequently found in postero-superior region suggesting an alternative impingement mechanism. Longitudinal studies investigating specific risk factors for hip injury will be beneficial by establishing causal links and stimulating effective preventative and treatment strategies.

**Table 1:** Search strategy

Search term	Hits between 01/01/1989 and 11/10/2021			Total
	Ovid	Embase	Cochrane reviews	
Hip* and (ballet or ballerina)	184	239	0	423
Hip*	374, 490	531,290	1746	907,526
Ballet or ballerina	1077	1473	0	2550

**Table 2:** Point prevalence of injuries sustained in ballet

Pathology	Study	Male:female ratio (*n*)	%	*n*
Ligamentum teres tears	Mayes et al. 2016a [54]	43% male 57% female	55.1	49 individuals
Ligamentum teres tears	Mayes et al. 2016a [54]	Male	52.4	21 individuals
Ligamentum teres tears	Mayes et al. 2016a [54]	Female	57.1	28 individuals
Hip joint effusion-synovitis	Mayes et al. 2020b [50]	43% male 57% female	44.9	49 individuals
Hip joint effusion-synovitis	Mayes et al. 2020b [50]	Male	38.1	21 individuals
Hip joint effusion-synovitis	Mayes et al. 2020b [50]	Female	50.0	28 individuals
ITB snapping hip	Winston et al. 2007 [82]	34% male 66% female	2.0	102 hips
Iliopsoas snapping hip	Winston et al. 2007 [82]	34% male 66% female	26.5	102 hips

**Table 3:** Incidence of injuries sustained in ballet

Injury	Study	Male:female ratio (*n*)	Prevalence (M:F)
Adductor muscle injury	Sobrino and Guillén, 2017 [72]	53% female 47% male (486)	0.007
Lateral snapping hip	Sobrino and Guillén, 2017 [72]	53% female 47% male (486)	0.005
Iliopsoas tendinopathy	Sobrino and Guillén, 2017 [72]	53% female 47% male (486)	0.004
Adductor tendinopathy	Sobrino and Guillén, 2017 [72]	53% female 47% male (486)	0.003
Anterior snapping hip	Sobrino and Guillén, 2017 [72]	53% female 47% male (486)	0.002
Hip synovitis	Sobrino and Guillén, 2017 [72]	53% female 47% male (486)	0.002
Gluteal/hip (including psoas) muscle spasm/strain/tear	Allen et al. 2012 [2]	Male (25)	0.13
Gluteal/hip (including psoas) muscle spasm/strain/tear	Allen et al. 2012 [2]	Female (27)	0.19
Groin tendinosis	Leanderson et al. 2011 [43]	62% female 38% male (476)	0.07

**Table 4:** Risk factors specific for hip injuries in ballet dancers

Study	Risk Factor	Pathology	Association
Sobrino and Guillén, 2017 [72]	Age	Lateral snapping hip	Higher rates of lateral snapping hip were found in junior professional dancers (≤ 21: 3.6%; 22–31: 3%; ≥ 32: 1.2%)
		Hip and pelvis pathology	Hip and Pelvis injury was more common in senior professional dancers (≤ 21: 13.4%; 22–31: 11.3%; ≥ 32: 22.4%)
	Sex	Hip pain injuries	Hip pain injuries are significantly more common in female dancers (*p* = 0.01)
Sobrino et al. 2015 [73]	Ballet discipline	Adductor Muscle Injury and Lateral Snapping Hip	Injuries of the adductor muscles of the thigh was most common in Spanish ballet (*p* = 0.001)
			Lateral snapping hip was more common in classical and Spanish ballet (*p* = 0.02)
Mayes et al. 2016a [54]	Hip anatomy, demographic and clinical parameters	Ligamentum Teres (LT) Tear	Those with an LT tear were older than those without (*p* = 0.004), and the severity increased with increasing age (*p* = 0.006) No difference in LCEA (*p* = 0.32, 0.16) or hip ROM (*p* > 0.01) between those with and without LT tears (low acetabular coverage is a known risk factor) Not associated with labral tears (*p* = 0.93, 0.03), cartilage defects (*p* = 0.09, 0.03), or BMI (*p* = 0.25)
Mayes et al. 2016b [52]	Hip anatomy, demographic and clinical parameters	Labrum Tear	No association between labral tear and hip ROM in 90 or 0 degrees of flexion Association between labral tear and cartilage defects was identified (*p* ≤ 0.001). Increasing age and cartilage defect presence were predictive for Labral tear development (*p* < 0.001) There was no difference in BMI (*p* = 0.57) or IPAQ (*p* = 0.78) between those with a labral tear and those without
Mayes et al. 2016c [53]	Hip anatomy, demographic and clinical parameters	Acetabular cartilage lesion	No association between sex (*p* = 0.45), BMI (*p* = 0.26) or IPAQ scores (*p* = 0.97) Age > 55 was significantly associated with cartilage defects in male dancers (*p* = 0.002)
Duthon et al. 2013 [19]	FAI and subluxation with normal anatomy in extreme ballet movements	Degenerative changes inc. labral tears, cartilage thinning and herniation pits	Degenerative changes including labral tears, cartilage thinning, and herniation pits, were located in superior and postero-superior positions in dancers. In controls, they tended to be found in the antero-superior position The authors suggest the position of these lesions may be due to repetitive extreme motions combining abduction and external rotation causing the femoral neck to abut the acetabular rim at this position during dance movements, despite normal anatomy The authors suggest that repetitive subluxations could be a cause of pain, and acetabular cartilage lesions as dancers hips showed a mean femoral head subluxation of 2.05 mm (﻿range 0.63–3.56 mm), in the splits position
Kolo et al. 2013 [39]	FAI and subluxation with normal anatomy in extreme ballet movements	Degenerative changes inc. labral tears, cartilage lesions and herniation pits	Degenerative changes were located differently between dancers and controls. Cartilage lesions predominantly were present at the superior position, and labral lesions were more pronounced lesions ﻿in the superior, postero-superior, and antero-superior positions, whilst herniation pits were frequently superior The authors suggest these lesions correlate with extreme positions achieved by the hip in ballet which are responsible for pincer-like impingement with linear contact between the superior or postero-superior acetabular rim and the femoral head–neck junction Authors suggest the loss of joint congruency observed contributes to cartilage stress and favors cartilage lesions
Charbonnier et al. 2011 [11]	FAI and subluxation with normal anatomy in extreme ballet movements	Degenerative changes and early hip OA	Ballet movements were optically tracked and the data applied to computed reconstructions of the joint. A high frequency of impingement was observed in the superior or postero-superior quadrant of the acetabulum, corresponding to the area at which degenerative lesions were found. Femoroacetabular translation during subluxation varied from 0.93 to 6.35 mm throughout the movements, and always correlated to an impingement, causing a loss of joint congruence and high labral stress The authors suggest that FAI and subluxation in the absence of cam or pincer morphological factors may lead to cartilage hyper-compression and be a potential factor for the development of hip OA
Mayes et al. 2018a [56]	Impingement-type osseous anatomy	Articular cartilage defects	Cartilage defect prevalence was higher in dancers with impingement-type bony morphology (﻿one of the following features: LCEA ≥ 39°, acetabular version < 10° or > 20°, alpha angle > 50.5° or NSA < 125°), compared to those without impingement-type morphology (*p* = 0.001) There was no relationship between instability-type (﻿one of the following features was detected: LCEA < 25° or NSA > 135°) bony morphology and cartilage defects (*p* > 0.05)
Blankenstein et al. 2020 [9]	Ballet participation	Anterior capsule thickening	Ballet dancers had a posterior capsule thickness higher than rugby playing controls (*p* = 0.03) and non-athletic controls (*p* = 0.03) The authors suggest that this is an adaptive focal physiological response to the ROM encountered at the hip joint
Hamilton et al. 2006 [30]	High-intensity dance training at 11–14 years	Femoral anteversion	﻿In the age range 11–14 years, those who trained more than six hours a week had less femoral anteversion (*p* = 0.02) The authors suggest that this may be an adaptive phenomenon to the increased mechanical loading during this critical period in growth
Mitchell et al. 2016 [59]	Osseous anatomy	Microinstability: femoral head subluxation in the splits manoeuvre	Subluxation occurs with a greater magnitude in women versus men as determined by vacuum sign prevalence on radiographs (*p* = 0.26) Subluxation magnitude increases with increasing alpha angle (*r* = 0.461, * p* = 0 .001) and decreasing NSA ﻿(* r* = − 0.332, * p* = 0 .022) In men, subluxation magnitude increases with severity of dysplasia (﻿lateral CEA * r* = − 0.437, * p* = 0.047; anterior CEA * r* = − 0.482, * p* = 0.027; ﻿Tönnis angle * r* = 0.656, * p* = 0.001; ﻿femoral head extrusion index * r* = 0.511, * p* = 0.018) In women, subluxation magnitude increases with decreased NSA (*r* = − 0.389, * p* = 0.049)
Assassi and ﻿Magnenat-Thalmann., 2016 [5]	Femoroacetabular impingement in extreme ballet movements	Degenerative changes inc. labral tears, cartilage lesions and herniation pits	Finite element modelling was applied to MRI data in the splits position. Strong deformations and pressures were observed during the simulation, with pressure peaks located in the posterior region, and contact area distributed between the infero-posterior and postero-superior regions. During the split posture there was a higher pressure and lower contact area than in daily activities These data suggest the repetitive extreme movements are sufficient to initiate degenerative changes in the acetabular cartilage and labrum
﻿Emery et al. 2019 [22]	Iliposoas cross-sectional area	HAGOS pain score	﻿Iliopsoas estimated marginal mean muscle CSA was 8% smaller in participants with hip pain compared to those with no hip pain (*p* = 0.035) Cross-sectional area of the muscle is related to strength. The authors suggest that reduced iliopsoas strength may lead to ﻿increased anterior hip joint forces and contribute to the development of hip pain or pathology Other hip flexors including TFL, sartorius and rectus femoris did not contribute to hip pain
Mayes et al. 2018b [51]	Obturator externus and internus cross-sectional area	HAGOS pain score	Neither muscle cross-sectional area was correlated to hip pain, indicating no effect of external rotator strength on hip pain in ballet dancers
Mayes et al. 2020a [58]	Bony morphology at baseline	Cartilage defects at five-year follow-up	Elite level ballet did not negatively affect cartilage health over 5 years, as the 10% progression observed here is very similar to that found in a prospective study scoring cartilage in the general population without signs of hip OA Cartilage defects were found solely in men. In men with cartilage defects, the femoral NSAs were lower (*p* = 0.004), indicating low NSA is related to premature cartilage degradation
Mayes et al. 2020b [50]	Demographic parameters and mobility	Hip joint effusion-synovitis	﻿Effusion-synovitis was not related to hip ROM, generalised joint hypermobility, or cartilage defect scores (P.0.05 for all) ﻿The prevalence of effusion-synovitis was similar in men (n = 11, 26%) and women (n = 24, 43%, P5 0.09) The prevalence of effusion-synovitis was similar between dancers (n = 22, 45%) and athletes (n = 13, 26.5%, * p* = 0.06) Symptomatic female dancers had a higher prevalence of effusion-synovitis (*p* = 0.002) and dancers with effusion-synovitis had a lower HAGOS pain (*p* = 0.001) and sports/recreation scores (*p* = 0.001)
Mayes et al. 2020c [57]	Hypermobility measured by Beighton 9-point score (≥ 5/9)	HAGOS pain score, cartilage defects on MRI and reported injuries	﻿Baseline and follow-up HAGOS pain scores were similar in GJH and non-GJH dancers (P.0.05 for all) At baseline ﻿Cartilage defect prevalence was lower in GJH (n 5 1) than non-GJH dancers (n 5 17, P, 0.001). At follow-up ﻿cartilage defects progressed in 2 dancers, one was hypermobile ﻿Hip-related injury over 5 years was reported by a similar number of GJH (n57) and non-GJH dancers (n5 6, P5 0.7)
Biernacki et al. 2020 [8]	Alpha angle measured by ultrasound	iHOT-12	Elite ballet dancers with an alpha angle > 60° had significantly lower iHOT-12 scores (73.4 ± 13.01) than those with alpha angles < 60° (80.22 ± 15.65; * p* = 0.001)
Emery et al. 2021 [21]	Deep hip external rotator muscle cross-sectional area	HAGOS pain score	Cross-sectional areas of piriformis, gemelli and quadratus femoris were not significantly associated with hip pain

**Table 5:** Outcomes for specific interventions reported in ballet dancers

Study	n	Ballet incidence	Intervention	Outcomes	Factors influencing outcomes
Ukwuani et al. 2019 [80]	F 62 M 2	Ballet dancers (66%)	Arthroscopy for FAI	97% returned to dance at an average of 6.9 ± 2.9 months. 62.5% returned to a better level of participation. 31% returned to the same level of participation. The number of hours danced per week decreased postoperatively (*p* = 0.041). Two patients were unable to return to dance, one with grade 4 chondromalacia and one who was involved in a road traffic accident	No differences were observed between the patient groups with GJL and without GJL (*p* > 0.1 for all outcomes) The number of years a patient had danced prior to surgery was moderately correlated with the time to return to dancing (r2 = 0.45, * p* = 0.001) Age, BMI, and level of competition had no correlation with return time (*p* > 0.05 for all)
Laible et al. 2013 [42]	F 43 M 6	Mixed cohort of ballet, modern, jazz or mixed dancers	Conservative treatment of iliopsoas syndrome. This consisted of activity specific rest, NSAIDs, and a comprehensive 12-week physical therapy programme focused on iliopsoas stretches, progressive iliopsoas strengthening, pelvic mobilisation, and antilordotic exercises	All 49 dancers had successful treatment, marked by a negative iliopsoas test and return to dance activity, without requiring escalation to corticosteroid injection or surgery	NA
Novais et al. 2018 [60]	F 33	Ballet and Modern dance	Periacetabular osteotomy (PAO) for hip dysplasia	63% (19/30) of females had returned to dance at an average of 8.8 months after PAO. There were improvements in mHHS (*p* = 0.01) and HOOS scores (*p* = 0.007)	No specific factors were associated with return to dance
